# Research on the Preparation and Anticorrosion Properties of EP/CeO_2_-GO Nanocomposite Coating

**DOI:** 10.3390/polym13020183

**Published:** 2021-01-06

**Authors:** Xiaoyan Liu, Handuo Jie, Ruidan Liu, Yanqi Liu, Tianyu Li, Kai Lyu

**Affiliations:** 1 College of Mechanics and Materials, Hohai University, Nanjing 210000, China; jiehanduo7@163.com (H.J.); liuruidan20@163.com (R.L.); yanqiliuniuniu@163.com (Y.L.); 187051500367@163.com (T.L.); 2 College of Civil and Transportation Engineering, Hohai University, Nanjing 210000, China

**Keywords:** CeO_2_, GO, CeO_2_-GO, dispersion, anti-corrosion performance

## Abstract

Due to its special two-dimensional lamellar structure, graphene possesses an excellent shielding effect, hydrophobic characteristics and large specific surface area, which can effectively isolate the internal structure from the external corrosive media. However, lamellar graphene is easy to stack and agglomerate, which limits its anti-corrosion performance. In this paper, cerium oxide-graphene oxide (CeO_2_-GO) nanocomposites were prepared by a hydrothermal synthesis method. Field emission scanning electron microscope (FESEM) and transmission electron microscope (TEM) were applied for microstructure examination, showing that a large number of nanoscale granular cerium oxide grew on the lamellar graphene oxide surface, which improved the dispersion performance of graphene inside the matrix. The anti-corrosion properties of the coating were analyzed and illustrated by open circuit potential (OCP), frequency response analysis, Tafel curve and Mott–Schottky curve. The results indicated that the CeO_2_-GO (4:1) nanocomposite not only eliminated the agglomeration of graphene to some extent, but also prepared the graphene epoxy coating with good dispersion, which further promoted its anti-corrosion performance. The paper proposed a feasible solution for GO dispersion in cement-based materials and lays a solid theoretical foundation for the engineering application of cerium oxide-graphene oxide modified anticorrosive coating.

## 1. Introduction

Metal is a widely used building structural material with excellent bending and tensile properties, while the metal corrosion is an unavoidable issue. Metal corrosion is the process in which metal materials are damaged by the action of surrounding media with the ingress of corrosion ions. In the process of corrosion, chemical or electrochemical reactions will occur at the interface of metal materials, which results in a significant degradation of strength, plasticity, toughness and other mechanical properties of metal materials, further destroying the geometric shape of metal components and shorten the service life of the structure. These leads to huge economic losses and energy waste. However, as an important part of construction materials, the metal materials are bounded to serve in the complex environments, such as acid rain [1] and salt [2]. According to previous research, the direct economic loss caused by metal corrosion is as high as 250 million us dollars every year [3,4]. Thus, corrosion protection of metal materials is of particular importance.

Surface coating is the most widely used protection method, among which epoxy coating is most commonly used for metal surface protection. Epoxy coating has excellent corrosion resistance, good mechanical strength and strong bonding performance of base material [5,6,7]. However, its long-term corrosion resistant performance is not outstanding and requires more effort [8]. As the erosion time increases, erosion medium will infiltrate through the micropores of epoxy coating and finally reach the surface of the mental substrate for sure [9]. In order to solve the above problems, nanocomposites are usually used to modify epoxy resin [10,11,12,13], so as to improve the insulation and shielding performance of epoxy coating.

Graphene (Gr) is an excellent two-dimensional nanomaterial with good stability and chemical inertness. As a kind of nanomaterial, mixing it into the epoxy coating will not change the original properties of the matrix. Graphene can not only block the passage of the smallest gas molecules, but also as a monatomic membrane, graphene not only blocks gas molecules, but also has a good blocking effect on the corrosion and dangerous ions [14]. Generally, graphene is not easy to be affect by the moisture [15,16]. The lamellar structure of graphene can provide good barrier properties for the coating. Therefore, through the design of graphene and its derivative graphene oxide (GO), a variety of methods were used to prepare GO composite material, and the composite coating was prepared by combining it with epoxy (EP) [17]. The excellent mechanical properties, chemical stability and shielding properties of GO improved the deficiency of EP coating [18,19]. In previous research, Lin et al. prepared GO microcapsules as a healing agent by means of self-assembly [20]. The addition of such microcapsules into the composite coating gives the coating self-healing properties and improves the corrosion resistance due to the physical barrier of GO shell. Zhang et al. [21] developed graphene composite anticorrosive materials based on lotus leaf effect, which repelled water and aqueous electrolytes to hinder the development of corrosion. Zhao et al. [22] developed an EP-Gr composite. According to Tafel curve analysis conducted during the full-immersion accelerated corrosion test, the corrosion current was reduced by at least 100 times compared to the pure and hydrophobic epoxy-coated chemically reduced sheets (CRS) sample. Xiu et al. [23] designed inhibitor coating on graphene composite material to passivate and thermally immunize base metal. However, due to the lamellar structure of graphene, the lamellar structure is easy to stack, which leads to the agglomeration of graphene. Therefore, it is necessary to improve its dispersion performance and prepare the graphene epoxy coating with good dispersion. Considering that cerium oxide (CeO_2_) has a nano-lamellar structure with certain barrier properties [24,25].

In this paper, cerium oxide-graphene oxide (CeO_2_-GO) nanocomposites with different CeO_2_ to GO mass ratios were prepared by hydrothermal synthesis method. The micromorphology was observed by field emitted scanning electron microscopy-energy dispersive X-ray spectroscopy (FESEM-EDS) and transmission electron microscopy (TEM). The anticorrosive properties of the coating were analyzed by open circuit potential test, frequency response analysis, Tafel curve and Mott–Schottky curve. The results are expected to promote the application of cerium oxide-graphene oxide modified anticorrosive coating in metal anticorrosion engineering.

## 2. Materials and Methods

### 2.1. Raw Materials

Graphene oxide (SE2430W) used in this research was a commercial product purchased from Changzhou Sixth Element Materials Technology Co., Ltd, Changzhou, China. The epoxy (WSR6101 E-44) and epoxy AB glue were supplied by Nantong Xingchen Synthetic Material Co., Ltd., Nantong, China. The epoxy resin (WSR6101 E-44) is a bisphenol A thermosetting epoxy resin with a viscosity of 15,000 mPa·s at 25 °C. The modified amine curing agent (593) is an addition of diethylenetriamine and butyl glycidyl ether with a viscosity of 90 to 150 mPa·s at 25 °C. Cerium hexahydrate nitrate ((CeNO_3_)_3_·6H_2_O) was analytical reagent from Shanghai Aladdin Bio-Chem Technology Co., Ltd., Shanghai, China. Ammonium hydroxide, ethyl alcohol absolute and acetone were both analytical reagents from Chengdu Colon Chemicals Co., Ltd., Chengdu, China. In the experiment, Q235 carbon steel with the size of 5 mm in height and 10 mm in diameter was selected to evaluate the anti-corrosion performance of the developed coatings.

### 2.2. Preparation of CeO_2_-GO Nanocomposites

**Figure 1:**
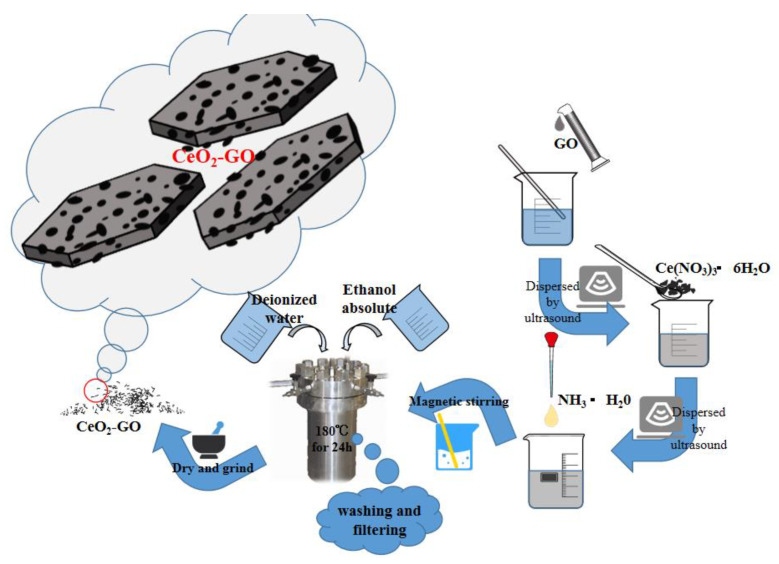
A schematic of synthesized process of cerium oxide-graphene oxide (CeO_2_-GO) nanocomposites.

The synthesized process of CeO_2_-GO nanocomposites is shown in Figure 1. Firstly, a certain amount of dialyzed GO slurry was weighed and dissolved in the water, and after the stripping process of an ultrasonic cell crushing machine, the thin-flake GO could be obtained. Then, an appropriate amount of cerium hexahydrate nitrate was added to the GO solution and ultrasonically stirred for 30 min, followed by another 30 min of magnetically stirring process, with the ammonia hydroxide added during the stirring process, for better dispersion. After that, the obtained solution was put in a high-pressure reaction kettle with 200 mL polytetrafluoroethylene liner mixed and reacted for 24 h at 180 °C. Finally, the solid phase within the solution was extracted by washing and filtering with deionized water and anhydrous ethanol [26], and after a drying and ground process, the CeO_2_-GO nanocomposite was produced. According to the above process, the CeO_2_-GO nanocomposite with mass ratios of CeO_2_:GO = 2:1, 4:1, 6:1 and 8:1 were prepared, respectively, for further CeO_2_-GO epoxy coatings manufacturing.

### 2.3. Preparation of CeO_2_-GO Epoxy Coatings

**Figure 2:**
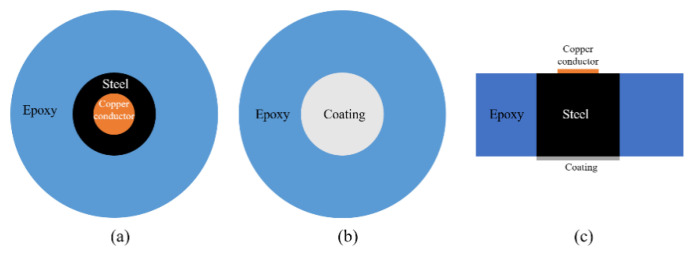
A schematic map of the prepared sample: (**a**) top view; (**b**) bottom view; (**c**) side view.

As aforementioned, a cylindrical Q235 carbon steel was applied to examine the anti-corrosion performance of the developed CeO_2_-GO coating. The sample was prepared with following procedures. Firstly, a copper conductor was affixed to the one side surface of the steel sample and the steel was centered into a cylindrical plastic mold (approximately 20 mm in diameter, 15 mm in height) with another side (no copper conductor attached) facing down. Then the AB epoxy was poured into the mold until it is roughly over the top surface of the steel sample and the mixture was kept in the mold for 24 h until the epoxy hardened. Then the sample was demolded, and the bottom surface of the sample was grinded and polished with abrasive papers in the sequential of 400, 800, 1000, 1200 and 2000 meshes with two purposes: (1) remove the excess AB epoxy resin and make the bottom of the steel sample exposed; (2) obtain a relatively smooth surface to improve the connection between the coating and steel sample. The sample was ultrasonically cleaned in alcohol to remove the debris caused by the grinding and polishing procedure. Then, 4 g of epoxy was measured and a certain amount of CeO_2_-GO (0.5 wt% of the epoxy mass) was added to the epoxy after a 30 min ultrasonic dispersion process. After that, the mixture was magnetically stirred for 30 min for better mixing and the acetone was removed by evaporation. Then, 1 g curing agent was added to the above mixture, and the mixture was stirred before it was stored in a vacuum drying oven to drive out the bubbles. Finally, the mixture was coated on the bottom surface of the Q 235 carbon steel with a coating machine and the coating thickness was controlled of 100 μm by a coating thickness gauge. The EP/CeO_2_-GO composite coating was obtained after cured at the room temperature for 24 h. For comparison, four samples denoted as G1, G2, G3 and G4, were prepared, with G1 being the pure epoxy coating and G2, G3 and G4, being the EP/CeO_2_-GO composite coating with CeO_2_ to GO mass ratios of 2:1, 4:1 and 6:1, respectively. A schematic map of how the sample was prepared is shown in Figure 2.

### 2.4. Characterization

#### 2.4.1. Micromorphological Characterization

The micromorphology and phase composition of CeO_2_-GO nanocomposites were characterized by FEI 3D field emission scanning electron microscope (Quanta FEG 250, FEI Co., Ltd., Hillsboro, OR, USA). The sample was thoroughly dried and grounded before it was sprinkled on a double-sided tape. The loose particles were blown off with the ear wash ball and the conductive tapes were ticked around the double-sided tape. After spraying gold, the sample was inserted into the mirror tube. The EDS analysis was coupled to accurately determine the elements of the composite sample.

In addition, JEM-2100 transmission electron microscope (Japan Electron Optics Laboratory Co., Ltd, Tokyo, Japan) was used to show the topography of how CeO_2_ particles grow inside the GO layers. As well, the sample was fully dried and ground, and then the appropriate amount of sample powder was taken and mixed evenly with ethanol, followed by an ultrasonic stirring process for 15 min. After stand still for 3–5 min, 2–3 drops of the mixture were drawn by a glass capillary tube and dropped onto a micro-grid for TEM observation.

#### 2.4.2. Electrochemical Testing

The electrochemical workstation of PARSTAT 2273 (Princeton Applied Research Co., Ltd., Oak Ridge, TN, USA) type was used for electrochemical testing, with the saturated calomel electrode being the reference electrode and platinum electrode as the auxiliary electrode. The electrolyte was the simulated concrete pore solution with 0.06 mol/L KOH, 0.2 mol/L NaOH, 0.01 mol/L Ca(OH)_2_ and 0.24 mol/L NaHCO_3_, and 0.6 mol/L NaCl was added as the erosion medium. The scanning range of open circuit potential (OCP) is −200 mV~1200 mV. The frequency range of EIS was 10^5^~10^−2^ Hz, and the amplitude was 10 mV. The scanning range of the potentiometric polarization curve was −300~300 mV, and the scanning rate was 1 mV/s. The adopted Mott–Schottky frequency was 1000 Hz, and the scanning interval was −1~0.5 V. The samples of G1, G2, G3 and G4 were tested.

## 3. Results

### 3.1. FESEM Analysis of Prepared Coating Composite

**Figure 3:**
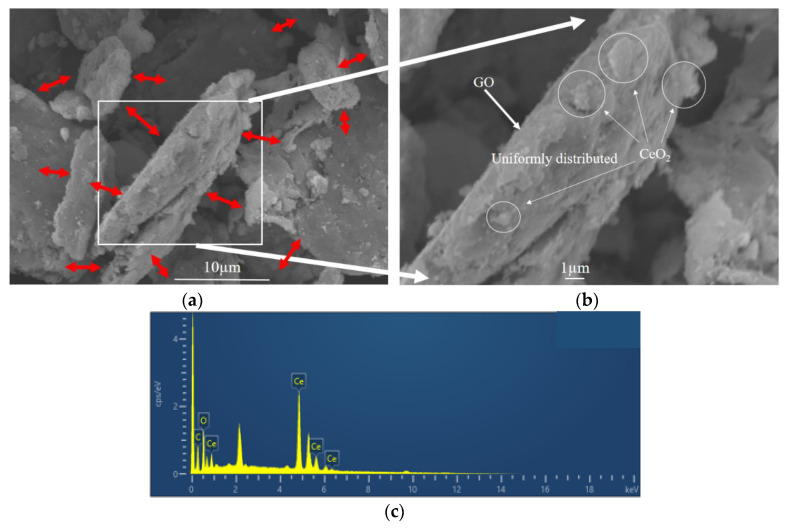
Micromorphological features of G3 (**a**,**b**) scanning electron microscopy (SEM) pattern; (**c**) energy dispersive X-ray spectroscopy (EDS) spectrum pattern.

The microstructure and elemental composition of CeO_2_-GO nanocomposites were analyzed by FESEM-EDS. For illustration, the G3 composite with CeO_2_ to GO mass ratio of 4:1 was selected. The microstructure of G3 composite is shown in Figure 3a,b, and the flake structure could be observed clearly and the CeO_2_ growing on GO tablets as indicated by the red arrows. A point was selected for energy spectrum analysis in Figure 3b. The EDS analysis showed that the surface of CeO_2_-GO was mainly composed of C, O and Ce elements, and the spectrum was shown in Figure 3c. The element line types and proportion of C, O and Ce are shown in Table 1. The weight percentage of Ce element and C element are 74.33%, and 16.05%, respectively, while the atomic percentage of C atom and Ce atom are 54.14%, and 21.49%, respectively. This may be attributed to that, on the surface of GO, there exists a large number of functional groups, such as carboxyl group and hydroxyl group, which provide active sites for the growth of Ce ions. In the process of hydrothermal synthesis, Ce(NO_3_)_3_·6H_2_O and NH_3_·H_2_O chemically reacted and granular CeO_2_ could grow at these active growth sites.

### 3.2. TEM Analysis of Best Mass Ratio of CeO_2_:GO

**Figure 4:**
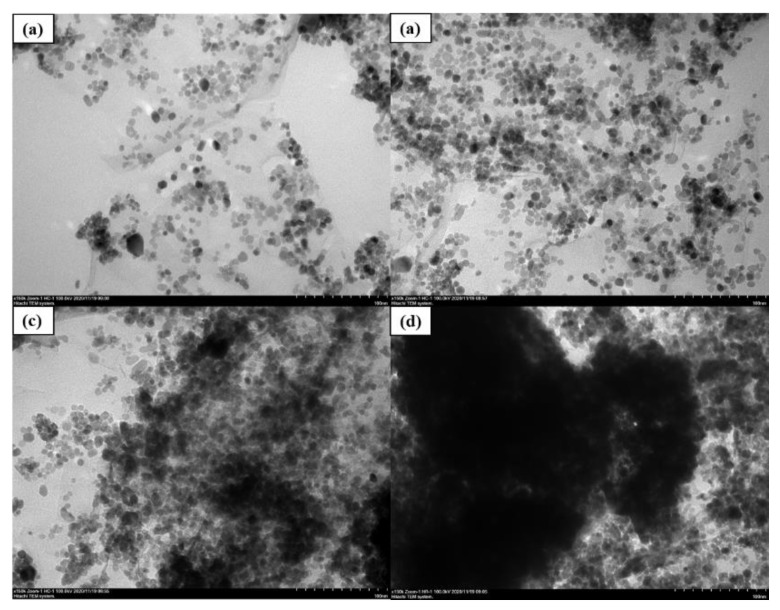
Micromorphological features of different mass ratio of CeO_2_-GO nanocomposites: (**a**) G2 SEM pattern; (**b**) G3 SEM pattern; (**c**) G4 SEM pattern; (**d**) G5 SEM pattern.

With our proper ultrasonic and magnetic mixing process, the GO nano-tablets were stretched with larger inter-layer spacing for nano-CeO_2_ growing and the nano-CeO_2_ particles could be seen evenly distributed on the GO layer surface. This finding indicated that that nano-CeO_2_ particles can not only solve the problem of GO lamination, but also solve the problem of the agglomeration of nanoparticles and obtain relative acceptable dispersion condition. To explore the mass ratio of the best combination of CeO_2_ and GO, four kinds of composite materials with different mass ratios of 2:1, 4:1, 6:1 and 8:1, denoted as G2, G3, G4 and G5, were prepared in this study. Figure 4 shows the micromorphological features of different mass ratios of CeO_2_-GO nanocomposites with TEM patterns. It can be all clearly observed from four images that CeO_2_ nanoparticles are growing on the surface of GO nanosheets to form CeO_2_-GO nanocomposites. While in Figure 4c,d, due to the excessive amount of CeO_2_ particle, there are insufficient place to be provided by the GO tablets for CeO_2_ particles growth, and CeO_2_ particles gradually agglomerate together, leading to be poor dispersion performance. Meanwhile, a relative better dispersion condition of CeO_2_ particles could be observed from Figure 4a,b. These both indicated a reasonable CeO_2_:GO mass ratio to be 2:1 and 4:1.

### 3.3. Open Circuit Potential (OCP) Analysis

**Figure 5:**
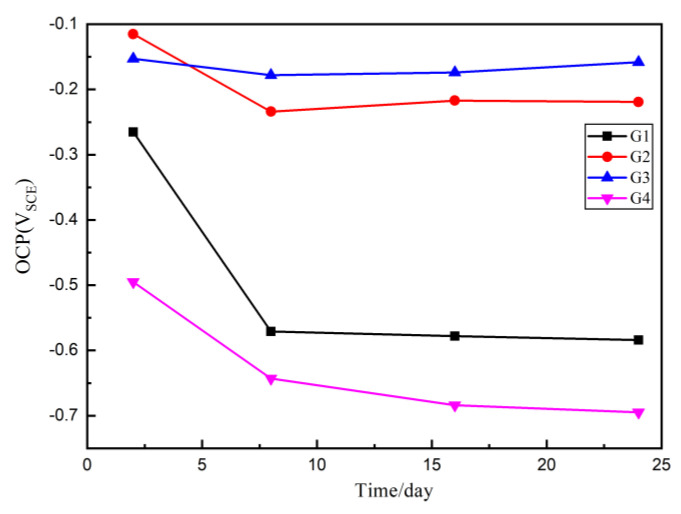
Each Epoxy (EP)/CeO_2_-GO coating’s open circuit potential (OCP) for immersion for 24 d.

According to American Society of Testing Materials standards (ASTM C876-91), when the steel bar self-corrosion potential is greater than or equal to −200 mV, the corrosion probability is 5%; when the steel bar self-corrosion potential is between −200 mV and −50 mV, the corrosion status is uncertain and the corrosion probability is 50%; when the self-corrosion potential of reinforcement is less than -350 mV, the corrosion probability is 95%. That means the higher the potential is, the lower the corrosion trend is, and the lower the potential is, the higher the corrosion trend is. Since in Section 3.2, where we proved that when the mass ratio of CeO_2_:GO is larger than 4:1, a poor dispersion of CeO_2_ particle was resulted, four coatings G1, G2, G3 and G4 were prepared, with G1 being the pure epoxy coating for comparison. The OCP results of four coatings eroded in the immersion for 24 h are shown and compared in Figure 5. Generally, up to 24 d, the OCP values of G2 and G3 are larger than G1 and G4, which proves that the composite has better barrier effect and provide better protection for the metal matrix. Further, the G2 and G3 OCP are greater than −200 mv for 2 d, indicating that there is no corrosion and the coating is relatively dense. However, the G4 OCP values are always smaller than 200 mv, indicating the corrosion occurred at the quite early stage, and this mainly due to a poor dispersion condition of CeO_2_ particles inside the GO layers, which further caused the galvanic corrosion and accelerated the corrosion. With the increase of immersing time, the potential of G4 and G1 decreases rapidly and the matrix is obviously corroded.

### 3.4. Electrochemical Alternating Current Impedance Spectroscopy (EIS) Analysis

**Figure 6:**
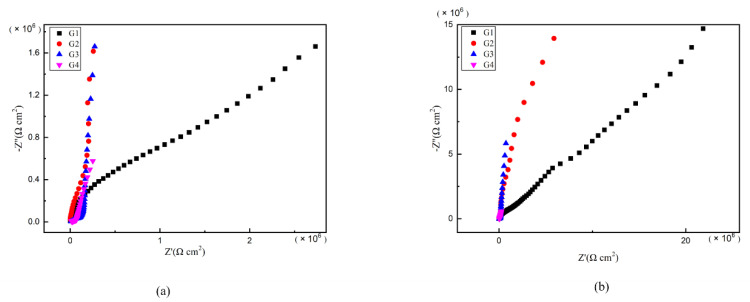
Each EP/CeO_2_-GO coating’s Nyquist pattern for immersion for 2 d: (**a**) Nyquist patterns for 2 d; (**b**) amplification of Nyquist patterns for 2 d.

**Figure 7:**
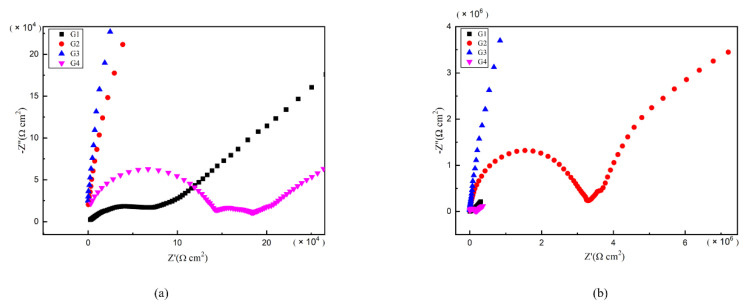
Each EP/CeO_2_-GO coating’s Nyquist pattern for immersion for 8 d: (**a**) Nyquist patterns for 8 d; (**b**) amplification of Nyquist patterns for 8 d.

**Figure 8:**
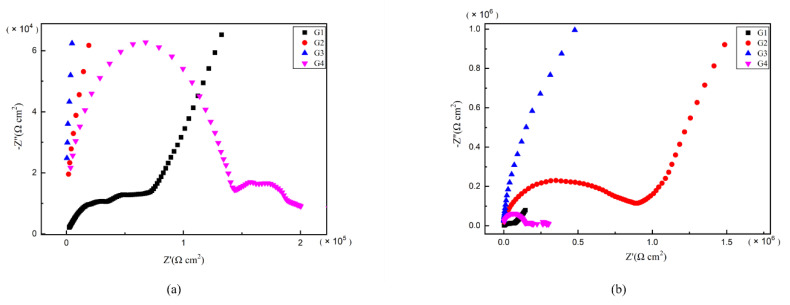
Each EP/CeO_2_-GO coating’s Nyquist pattern for immersion for 16 d: (**a**) Nyquist patterns for 16 d; (**b**) amplification of Nyquist patterns for 16 d.

**Figure 9:**
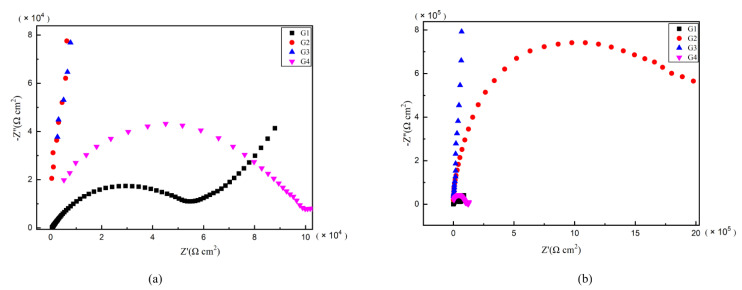
Each EP/CeO_2_-GO coating’s Nyquist pattern for immersion for 24 d: (**a**) Nyquist patterns for 24 d; (**b**) amplification of Nyquist patterns for 24 d.

As is well known, electrochemical alternating current impedance spectroscopy (EIS) tests can be used to evaluate the corrosion resistance of coatings and to study the evolution of impedance models during coating failure [27] and this method was applied in this study. Figure 6, Figure 7, Figure 8 and Figure 9 show Nyquist patterns of carbon steel coated with different coatings immersed in erosion solution for 2 d, 8 d, 16 d and 24 d, respectively. It can be seen from Figure 6 that when immersion time was 2 d, the arc radius of each coating was larger, showing that the barrier property of the coating was intact and no erosion medium passed through the coating to reach the metal substrate. With the extension of immersion time, the arc radius of each coating decreases continuously. When the immersion time is 8 d, the arc radius of G4 and G1 decreases significantly, indicating that the corrosive medium has penetrated into the surface of carbon steel and corrosion begins to occur. Due to poor dispersion and excellent electrical conductivity of graphene, the barrier effect of G4 as an anticorrosive coating is far less than the influence of electric couple corrosion brought by its electrical conductivity. Thus, its protection efficiency was weakened. G3 has the largest capacitance-reactance radius, and is slower than G2 as the immersion time decreases, showing an excellent anti-corrosion performance.

**Figure 10:**
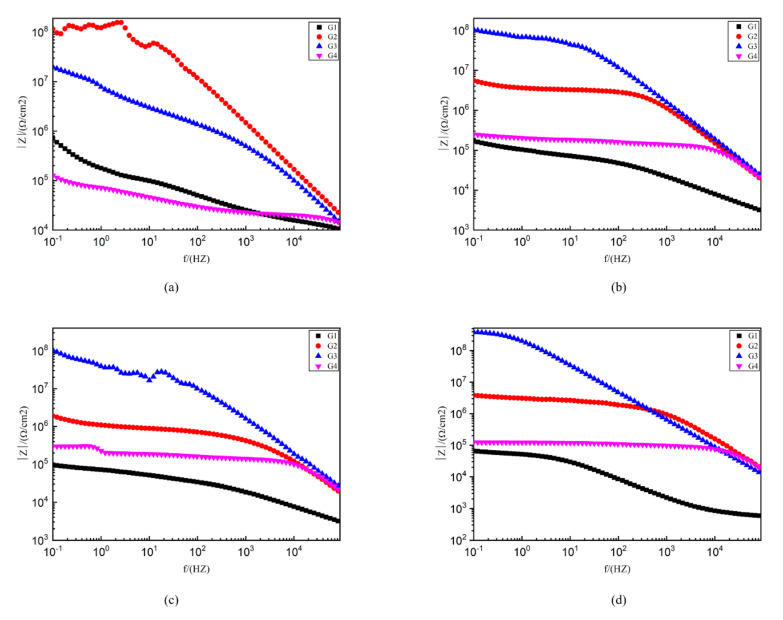
Each EP/CeO_2_-GO coating’s Bode pattern for immersion for 2 d, 8 d, 16 d and 24 d, respectively: (**a**) Bode pattern for 2 d; (**b**) Bode pattern for 8 d; (**c**) Bode pattern for 16 d; (**d**) Bode pattern for 24 d.

Figure 10 shows the Bode pattern of each coating immersed for 2 d, 8 d, 16 d and 24 d. The modulus value of low-frequency area in the Bode pattern can reflect the protective performance of the coating. The higher the modulus value of low-frequency area is, the better the protective performance of the coating is [28]. G1 |Z|0.01 HZ value from 4.63 × 10^9^ Ω/cm^2^ (2 d) down to 9.72 × 10^4^ Ω/cm^2^ (24 d) shows that the EP coating anticorrosive ability as the extension of immersion time decreases obviously, and the protection ability is weak. G4 |Z|0.01HZ value from 6.3 × 10^5^ Ω/cm^2^ (2 d) down to 1.32 × 10^5^ Ω/ cm^2^ (24 d). Although the extent of the decline is lesser, excessive CeO_2_ composite materials are in the EP reunion. At the same time, the existence of galvanic corrosion reduces G4 impedance modulus value and anti-corrosion ability. G2 and G3, by contrast, the |Z| 0.01HZ value is higher. G2 24 d |Z| 0.01HZ value at 10^7^ Ω/cm^2^ and G3 24 d |Z| 0.01HZ value as high as 10^8^ Ω/cm^2^ showed good anticorrosion performance.

The uniform dispersion of fillers in the coating plays an important role in the corrosion resistance of the coating [29]. The modification of GO by nano CeO_2_ particles improves the agglomeration of GO in the coating. At the same time, the problems of easy accumulation of nanoparticles itself improves a lot. The synergistic effect of CeO_2_ and GO enhances the blocking effect of fillers in coatings. Thus, the corrosion resistance of epoxy coating is greatly improved.

### 3.5. Tafel Curve Analysis

**Figure 11:**
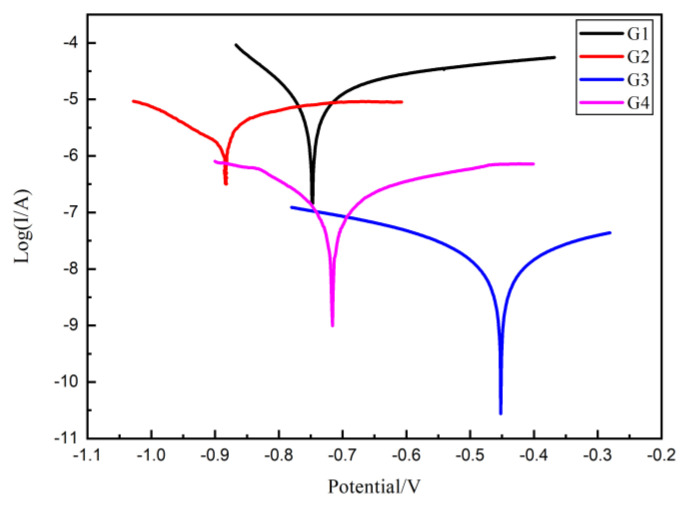
Each EP/CeO_2_-GO coating’s Tafel curve for 160 d.

The protective performance of the developed epoxy coating was further analyzed by the Tafel curve and the results of each coating immersed for 160 d are compared in Figure 11. It can be seen from Figure 11 that G3 has the minimum corrosion current density, while G1 has the maximum corrosion current density. The difference between the two is three orders of magnitude. The protection efficiency (*η*) of the coating can be calculated from the following equation:

(1) $$\eta \%=(1-I_{\mathrm{corr}}/I_{\mathrm{corr}0})\times 100\%$$

where *Icorr0* represents the corrosion current density of pure EP coating, *Icorr* represents the corrosion current density of the certain anticorrosive coating and *η* represents the protective efficiency of coating.

The polarization resistance (*R_p_*) of the coating can also be obtained by Tafal curve, which is calculated by Equations (2) and (3), where *b_a_* and *b_c_* are the Tafel slope of the anode and cathode, respectively, which can both be obtained from linearly fitting the Tafel curve.

(2) $$B=b_{a}b_{c}/2.303\left(b_{a}+b_{c}\right)$$

(3) $$R_{p}=B/I_{\mathrm{corr}}$$

Thus, for four kinds of coatings, the corrosion potential (*E_corr_*), corrosion current density (*I_corr_*), polarization resistance (*R_p_*) and protection efficiency (*η*) in 160 d are listed and compared in Table 2. Compare with G1, the *R_p_* of other three EP/CeO_2_-GO all much smaller, indicating an increased protection effect. Obviously, the corrosion current density of G3 is the lowest, only 1.276 × 10^−8^ A/cm^2^, and the polarization resistance is the highest, reaching 3.463 × 10^6^ A/cm^2^, which is 3 orders of magnitude higher than that of G1, accompanying a highest protection efficiency of 99.88%, which is significantly superior than other coatings.

### 3.6. Mott–Schottky Curve Analysis

**Figure 12:**
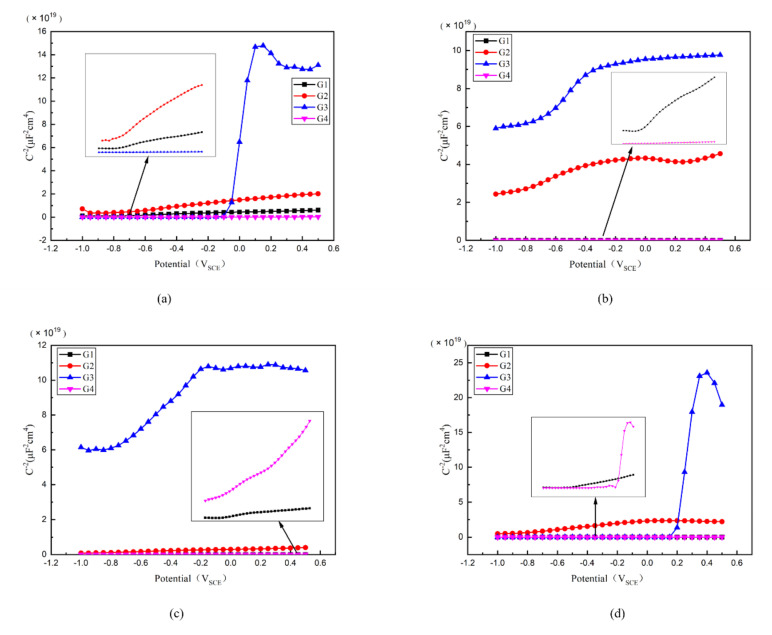
Each EP/CeO_2_-GO coating’s Mott–Schottky pattern for immersion for 2 d, 8 d, 16 d, 24 d: (**a**) Mott–Schottky pattern for 2 d; (**b**) Mott–Schottky pattern for 8 d; (**c**) Mott–Schottky pattern for 16 d; (**d**) Mott–Schottky pattern for 24 d.

Then, the Mott–Schottky curve was introduced for analysis and the Mott–Schottky for all kinds of coatings immersed for different ages were compared in Figure 12. Particularly, the Mott–Schottky curve slope of each coating is positive when immersed in 2 d, which conforms to n-type semiconductor property [30]. For G3, it has the highest slope and the lowest carrier density, as well as the least electron migration and ion penetration occurring within the coating. The slope of G2 is greater than that of G1 and G4, meaning G2 has a relatively smaller carrier density. While for G4, the slope of Mott–Schottky curve is less than that of G1, and could be attributed to the fact that excessive CeO_2_ grows between the GO layers, which leads to the stacking of GO, resulting in the galvanic corrosion. This effect weakens the anticorrosion performance of coating.

The carrier density of each coating changing with immersion time is compared in Table 3. Generally, with the extension of immersing time, the carrier density of G1 and G4 gradually increases, and the erosion medium continuously penetrates into the coating. The corrosion worsens continuously. However, the carrier density of G3 and G2 increased slowly and roughly remained at the order of 10^8^~10^9^ even until 24 d. Considering this finding, the water may play an important role. Where with water infiltrated into the coatings, the infiltration rate decreased with the increasing internal moisture of the coating. Due to the water-solubility of CeO_2_ nanoparticles, this further blocks the ingress of the erosion media to some extent. This leads to a weakened carrier density of ions for G2 and G4, which is proved by the fact that the 24 d carrier density of G3 is only 7.577 × 10^8^ cm^−3^, further strengthening the anti-corrosion performance of G2 and G3 epoxy coatings.

## 4. Discussion

According to the experimental results and analysis, CeO_2_ particles can grow evenly between the GO layers, effectively solving the stacking and agglomeration problems of the layers. In this paper, CeO_2_-GO nanocomposites were prepared by hydrothermal synthesis method, and the nanocomposites with different CeO_2_ to GO mass ratios were prepared. It was found that a large amount of nano-scale granular CeO_2_ grew on the flake GO surface by FESEM-EDS and TEM observations. GO is widely considered to be hydrophilic, but due to the abundant functional groups between the layers, it can provide abundant sites for synthesis. The hydrophilicity of GO is related to its hydrophilic group, and the combination of a large amount of CeO_2_ with GO can reduce the active sites of GO, which can weaken its hydrophilicity and also improve the stacking of GO sheets. The anti-corrosion properties of the coating were analyzed by OCP, EIS, Tafel curve and Mott–Schottky curve. For common epoxy coating, when the corrosion ions penetrated into the coating, no agents could protect the penetration process of corrosion ions and they could reach the surface of steel bar directly. For the coating G2 (CeO_2_:GO = 2:1), the GO was relatively evenly dispersed inside the epoxy coating with incorporation of CeO_2_ particles, which help to block the ingress of corrosion ions, while water-solubility CeO_2_ strengthened this blocking effect. Commonly, as the immersion time increases, water and other erosive ions continue to penetrate through the micro-pores in the coating, while CeO_2_ grows between the GO layers, making GO well dispersed in the epoxy resin, reducing the stacking folds of its layers and making the penetration path of the erosive medium more complex and tortuous. At the same time, the invading water reacts with Ce ions to produce Ce(OH)_4_ precipitate, which expands slightly in volume and blocks in the micro-pores, preserving the integrity of the coating to a certain extent and slowing down the infiltration rate of the erosion medium. Overall, considering the TEM, SEM and electrochemical tests, the recommended CeO_2_:GO mass ratio was 4:1. At this ratio, the GO layers could provide enough space for CeO_2_ growth, and no excessive CeO_2_ particles agglomeration occurs. Compared with G2, the blocking effect brought by GO was certain, while role of CeO_2_ particles was enlarged since more CeO_2_ particles were included in G3 compared with G2. However, when increasing CeO_2_ to GO mass ratio, excessive CeO_2_ particles agglomerated inside the GO layers with a poor dispersion performance, which weakens the blocking effect.

## 5. Conclusions

In this paper, CeO_2_-GO nanocomposites with different mass ratios were prepared by hydrothermal synthesis method. The anticorrosion performance of EP/ CeO_2_-GO coatings was analyzed by electrochemical means. The following conclusions can be reached based on experimental evidence.

(1) The SEM and TEM analysis indicated that via hydrothermal synthesis, GO was more effectively dispersed and the inter-layer inside the GO could provide space for CeO_2_ growth. The recommended CeO_2_ to GO mass ratio was 4:1, while with increasing ratios, GO and CeO_2_ would agglomerate inside the GO layers.
(2) The anticorrosion properties of CeO_2_-GO nanocomposites as coatings were analyzed by OCP, EIS, Tafel curve and Mott–Schottky curve. Tafel curve showed that CeO_2_-GO (4:1) had the lowest corrosion current density and the highest polarization resistance. The protective efficiency of the coating was up to 99.88%. The Mott–Schottky curve showed that the 24 d carrier density of CeO_2_-GO (4:1) was only 7.577 × 10^8^ cm^−3^, showing an excellent anticorrosion effect.
(3) This study found that CeO_2_-GO (4:1) nanocomposite can not only improve the agglomeration of graphene, but also prepare graphene epoxy coating with good dispersion. The anticorrosion effect was very significant. That is beneficial to promote the engineering of CeO_2_-GO modified anticorrosive coating and improve the corrosion of metal.

## Figures and Tables

**Table 1:** Element line type and proportion.

Element	Line Type	Weight Percentage	wt% Sigma	Atomic Percentage
C	K line series	16.05	0.45	54.14
O	K line series	9.63	0.29	24.37
Ce	L line series	74.33	0.49	21.49
Total	/	100.00	/	100.00

**Table 2:** Kinetic parameters of each coating.

Coating	*E_corr_* (V)	*I_corr_* (A/cm^2^)	*η* (%)	*R_p_* (ohm)
G1	−0.747	1.079 × 10^−5^	/	3.222 × 10^3^
G2	−00.893	2.814 × 10^−6^	73.92	8.410 × 10^4^
G3	−00.451	1.276 × 10^−8^	99.88	3.463 × 10^6^
G4	−00.7153	1.605 × 10^−7^	98.51	2.550 × 10^5^

**Table 3:** Each EP/CeO_2_-GO coating’s carrier density for 2 d, 8 d, 16 d, 24 d.

Coating	2 d ND (cm^−3^)	8 d ND (cm^−3^)	16 d ND (cm^−3^)	24 d ND (cm^−3^)
G1	2.501 × 10^10^	1.045 × 10^13^	3.679 × 10^13^	3.550 × 10^14^
G2	7.403 × 10^9^	6.740 × 10^9^	4.296 × 10^10^	6.229 × 10^9^
G3	7.853 × 10^8^	2.988 × 10^9^	2.268 × 10^9^	7.577 × 10^8^
G4	7.373 × 10^11^	1.048 × 10^13^	5.061 × 10^12^	1.030 × 10^14^

## Data Availability

The data presented in this study are available on request from the corresponding author.
